# Enteric Microbiota-Mediated Serotonergic Signaling in Pathogenesis of Irritable Bowel Syndrome

**DOI:** 10.3390/ijms221910235

**Published:** 2021-09-23

**Authors:** Yoshiyuki Mishima, Shunji Ishihara

**Affiliations:** Department of Internal Medicine II, Shimane University Faculty of Medicine, Izumo 693-8501, Japan; si360405@med.shimane-u.ac.jp

**Keywords:** SERT, TPH, enterochromaffin cell, quorum sensing, colitis, mucosal immunology, enteric nervous system, central nervous system

## Abstract

Irritable bowel syndrome (IBS) is a chronic functional disorder that affects the gastrointestinal tract. Details regarding the pathogenesis of IBS remain largely unknown, though the dysfunction of the brain-gut-microbiome (BGM) axis is a major etiological factor, in which neurotransmitters serve as a key communication tool between enteric microbiota and the brain. One of the most important neurotransmitters in the pathology of IBS is serotonin (5-HT), as it influences gastrointestinal motility, pain sensation, mucosal inflammation, immune responses, and brain activity, all of which shape IBS features. Genome-wide association studies discovered susceptible genes for IBS in serotonergic signaling pathways. In clinical practice, treatment strategies targeting 5-HT were effective for a certain portion of IBS cases. The synthesis of 5-HT in intestinal enterochromaffin cells and host serotonergic signaling is regulated by enteric resident microbiota. Dysbiosis can trigger IBS development, potentially through aberrant 5-HT signaling in the BGM axis; thus, the manipulation of the gut microbiota may be an alternative treatment strategy. However, precise information regarding the mechanisms underlying the microbiota-mediated intestinal serotonergic pathway related to the pathogenesis of IBS remains unclear. The present review summarizes current knowledge and recent progress in understanding microbiome–serotonin interaction in IBS cases.

## 1. Background

Irritable bowel syndrome (IBS), a gastrointestinal (GI) disorder characterized by chronic abdominal pain with aberrant bowel movements in the absence of nondetectable causes [1,2], is the most common GI disease, with global prevalence estimated to be 10–15% [3]. Rome IV criteria are commonly used to diagnose this disorder in clinical practice, which classify cases into four different subtypes on the basis of bowel habits and fecal condition: constipation-predominant (IBS-C), diarrhea-predominant (IBS-D), mixed (IBS-M), and unclassified (IBS-U) [4,5].

Details regarding the pathogenic mechanisms remain largely unclear; thus, current treatments for IBS are mostly focused on symptoms, with limited efficacy, and not as radical care [6,7,8]. Although this disorder is not associated with increased risk of mortality [9], a number of IBS patients show markedly decreased quality of life, including poor socioeconomical activities as a result of IBS [2,10]; thus, the high prevalence of chronic features in affected patients places a financial burden on global healthcare systems [11,12,13]. As a result, the introduction of an effective treatment strategy based on mechanistic studies is highly anticipated.

Numerous basic and clinical studies were conducted to clarify the cause of IBS from multiple aspects, including genetic factors, low-grade mucosal inflammation and immune activation following a severe GI infection, increased gut mucosal permeability, alterations in gut microbiota, aberrant bile salt metabolism, hypersensitivity to particular diet components, abnormal neurotransmitter pathways, and altered central nervous system (CNS) processing [14,15,16,17,18]. Although each factor contributes to forming a certain portion of the IBS etiology, clinical features of the disorder are heterogeneous, and likely created by a mixture of genetic and environmental factors [15,16]. Therefore, the determination of and focus on a specific target molecule or pathway related to IBS pathogenies is challenging, though recent progress in omics technologies, including epigenomics, metabolomics, transcriptomics, and proteomics, provided efficient methods for identification of new pathways and potential targets [19,20,21]. These novel approaches provided results suggesting the importance of interactions between enteric microbiota and neurotransmitters, and their pathways in the context of IBS pathogenesis [15,18,22,23].

Neurotransmitters are chemical substances that transmit signals between neurons and target cells, such as muscles, glands, and other neurons, throughout the body [24]. Enteric microbiota play a critical role in regulating a variety of neurotransmitters, including histamine, serotonin (5-hydroxytryptamine, 5-HT), glutamate, γ-aminobutyric acid (GABA), dopamine, acetylcholine, and catecholamines [18,22,24], and each biological substance influences the activity of the enteric nervous system (ENS) independently or corporately as part of the pathology of IBS [22,25]. Among those, 5-HT is one of the most well-studied neurotransmitters in IBS research investigations [26,27,28]. Enteric 5-HT boosts visceral hypersensitivity, increases mucosal permeability, alters gut motility, activates the immune system, and induces inflammation, which synergistically contribute to forming IBS symptoms [28]. Indeed, pharmacological interventions regarding 5-HT receptors are commonly used for treating IBS patients in current clinical practice [7,29]. Overall, 5-HT in association with enteric microbiota is a promising target for both medical care and IBS research.

Nevertheless, unanswered questions remain before fully revealing the etiology of IBS because of insufficient evidence, likely due to abundant limitations in IBS research [22,30] because it is often difficult to acquire quantified reproducible data when investigating functional disorders such as IBS for clinical and preclinical studies. Clinical studies of IBS patients tend to show huge placebo effects, which make the obtained results difficult to interpret [1,31]. Unlike other GI disorders, such as inflammatory bowel disease (IBD), there are neither reliable tests nor universally accepted biomarkers available to diagnose and evaluate IBS-related disease activity [5,30,32]. In preclinical studies, several animal IBS models that show abnormal intestinal motility and/or visceral hypersensitivity have been established. However, those are completely different from human IBS in terms of dietary contents and habits, social behavior, mental status, the intestinal immune system, and resident enteric microbiota [30,33,34]. Additional technology and knowledge for dealing with these limitations are needed.

This review summarizes existing evidence and recent progress in the involvement of gut microbiota-mediated 5-HT in IBS. Additional research targeting the interactions between enteric microbiota and serotonergic signaling could provide deeper understanding and mechanistic insight regarding the pathology of IBS.

## 2. Brain–Gut–Microbiome Interactions in IBS

Trillions of gut microbes coexist in humans and supply a variety of beneficial functions to the host, such as creating essential nutrients and vitamins from indigestible or poorly absorbable dietary contents, training the immune system, and limiting the settlement or growth of harmful microorganisms [35,36,37]. An alternation of the gut microbial population, termed dysbiosis, is potentially associated with both GI and non-GI disorders, such as diabetes, obesity, chronic kidney disease, and several psychiatric and neurologic disorders [38,39,40]. Unfortunately, the underlying causes and mechanisms of dysbiosis in related diseases are largely unknown. In fact, it is yet to be shown whether dysbiosis is a cause or consequence of a target disease, while even a “healthy microbiome” is poorly defined, which is a million-dollar question [41,42,43].

Numerous research studies were conducted to investigate mechanisms related to the influence of the gut microbiota on health and disorders beyond the GI tract, particularly in the brain [44]. Some preclinical and clinical studies showed that gut microbes communicate with the CNS through multiple channels, including nervous, endocrine, and immune signaling pathways [23,45,46]. Additionally, the brain influences the intestinal microenvironment by modulating gut motility, secretion, and permeability through the neuron–glia–epithelium axis and visceral nerves [23,44,45,46]. On the basis of these findings, the concept of the brain–gut–microbiome (BGM) axis was proposed to elucidate bidirectional communication between gut microbiota and the CNS [23,45,46].

The dysfunction of BGM interactions is a central pathological factor in the context of IBS [22,23,45,46], and dysbiosis was observed in patients with IBS in most related clinical studies [47,48,49]. Traditional fecal analysis results demonstrated a certain bacterial population in IBS patients that is distinctively different from that in healthy individuals, with lower bacterial diversity [40,50,51,52,53]. Fecal samples from IBS patients show a higher *Firmicutes/Bacteroidetes* ratio, lower abundance of *Lactobacillus* and *Bifidobacterium*, and higher levels of *Escherichia coli* and *Enterobacter* [30,54,55,56,57]. In addition, *Clostridiales* I, *Faecalibacterium*, and *Bifidobacterium* genera were reported to be decreased in IBS patients [58]. Recently, the presence of mucosal biofilm consisting of an overgrowth of *E. coli* and *Ruminococcus gnavus* was proposed to be an endoscopic feature in a subgroup of IBS patients [59]. That study noted that such biofilms contain high concentrations of bile acids that can induce bile acid-dependent abnormalities in bowel movements [59]. These observations suggest that reversing the altered bacterial composition, along with normalizing the BGM axis, may be an ideal treatment strategy for IBS, though it is unknown if dysbiosis has a causal effect for IBS development. Moreover, though bacteria are the richest and most well-investigated intestinal microorganisms, dysbiosis is also found in viral or fungal compositions in IBS patients. Multiomics analyses indicated specific IBS subset-related changes in phage populations [19], while mycobiome analysis findings revealed that intestinal fungi play an important role in the pathogenesis of IBS [60]. Observations from these different viewpoints profoundly indicate the complexity of enteric microbiology and encountered difficulties in microbiology research.

Epidemiological evidence showed that psychiatric and GI functional disorders are frequently complicated [61,62]. Individuals suffering from anxiety or depression have a significantly high prevalence of IBS, while IBS patients have a threefold increased risk of anxiety or depression as compared with that of healthy controls [61,62,63]. Some drugs developed primarily for psychiatric disorders can also be effective in IBS patients, and change the gut microbial composition [64,65,66]. In addition, microbial dysbiosis with aberrant microbial metabolites and ENS dysfunction are related to a variety of neurological and psychiatric disorders, including autism spectrum, Parkinson’s disease, Alzheimer’s disease, anxiety, and depression [23,67,68,69].

The microbiota also plays a significant role in CNS and ENS development [68,69]. Germ-free (GF) animals and rodent models treated with broad-spectrum antibiotics in early life had abnormal neurodevelopment [70]. GF mice showed increased permeability of the blood–brain barrier (BBB) with an immature phenotype of CNS microglia, which was reversed by fecal transplantation from specific pathogen-free (SPF) mice or the administration of bacteria producing short-chain fatty acids (SCFAs) [71]. Additionally, GF mice had disrupted motor activity with reduced anxiety-like behavior due to the altered expression of anxiety and synaptic-plasticity-related genes in the brain as compared to mice with a normal intestinal microbiota [72]. On the other hand, microbiota-derived molecules or metabolites can induce host-derived cytokines and inflammation in the CNS, which contribute to development of brain disorders by changing BBB permeability, brain vascular physiology, and brain structure [44]. Increased numbers of *Akkermansia muciniphila* and *Acinetobacter calcoaceticus*, and a decrease in *Parabacteroides distasonis* were observed in patients with multiple sclerosis [73,74], while several dysbiosis-related conditions in Parkinson’s disease were also reported [75]. These results indicate the importance of the quality of resident microbiota to maintain homeostasis in the gut and brain.

Together, these findings show that, although it remains unclear why and how a dysfunction of the BGM axis emerges, breaking the vicious cycle of aberrant BGM interactions by modulating gut microbiota may be an effective treatment option for patients with IBS and psychiatric disorders [55,76,77]. Indeed, antibiotics and probiotics were effective for some IBS patients, with fecal microbial transplantation (FMT) currently being investigated in clinical studies [50,78,79,80]. Reports of clinical trials based on evidence obtained from systematic microbial research results are anticipated.

## 3. Microbiota-Mediated Serotonergic Signaling in Intestines

**Figure 1:**
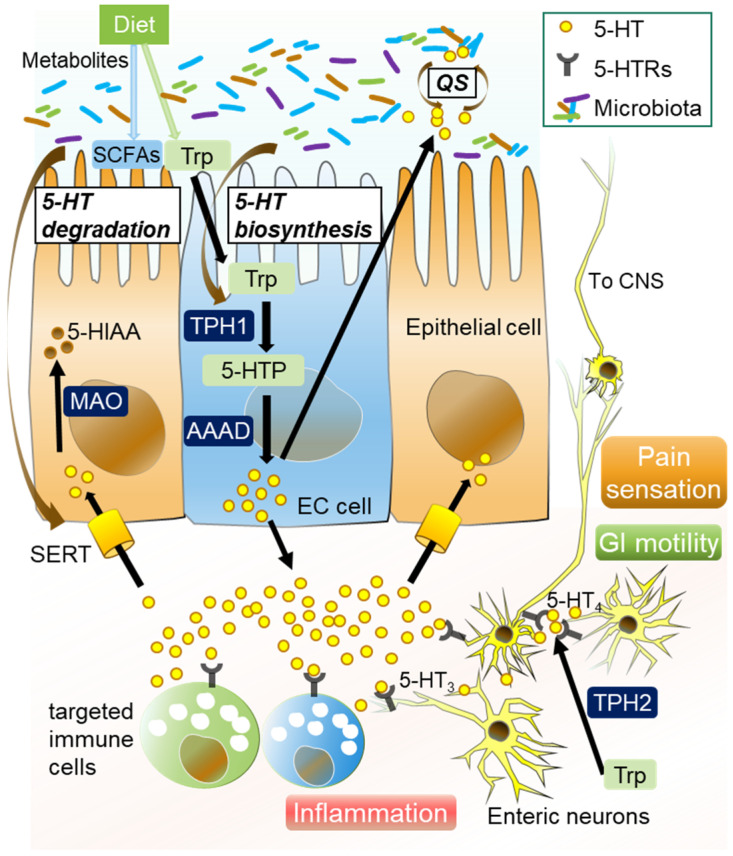
Overview of serotonergic pathway modulated by gut commensal microbiota components. Gut 5-HT is biosynthesized with L-tryptophan (Trp) derived from dietary contents in enterochromaffin (EC) cells, in which tryptophan hydroxylase 1 (TPH1) converts Trp into 5-hydroxytryptophan (5-HTP), a direct precursor of 5-HT. Activation of EC cells and TPH1 is dependent on resident microbiota and their metabolites. Released 5-HT binds to various 5-HT receptors (5-HTRs) on immune cells, such as mast cells and macrophages, which potentially induces mucosal inflammation in the gut. Enteric 5-HT can modulate GI motility by stimulating the enteric nervous system (ENS), while it also acts to induce pain by activating afferent fibers as well as inducing mucosal inflammation, mainly through 5-HT_3_ and 5-HT_4_ receptors. Released 5-HT is taken up by serotonin reuptake transport (SERT) and degraded by monoamine oxidase (MAO) into 5-hydroxyindole acetic acid (5-HIAA) in the cells to optimize the luminal 5-HT level. 5-HT can also influence bacterial composition by quorum sensing (QS).

Enteric 5-HT plays a significant role in the BGM axis under both homeostatic and pathogenic conditions [81]. Over 95% of total body 5-HT exists in the GI tract, where it is biosynthesized with L-tryptophan, mainly in enterochromaffin (EC) cells [82,83]. GF and antibiotics-treated mice displayed significantly low levels of peripheral 5-HT [84,85], while colonization with normal gut microbes increased colonic 5-HT production [81]. Specific resident bacteria, including *Streptococcus* spp., *Enterococcus* spp., and *Corynebacterium* spp., directly produce 5-HT [86], while a specific enteric resident microbiota modulates host peripheral serotonin levels. Indigenous spore-forming bacteria and *Clostridium ramosum* promote 5-HT biosynthesis from colonic EC cells [85,87]. *A. muciniphila* and its extracellular vesicles promote intestinal 5-HT biosynthesis and extracellular availability through TLR2 signaling [88,89], while SadA-expressing Staphylococci promote peripheral 5-HT synthesis [90]. Another study showed that probiotic strain *Escherichia coli* Nissle *1917* enhanced 5-HT bioavailability in ileal tissue [91], while *Lactobacillus rhamnosus* exerted antidepressant effects and decreased colonic 5-HT levels in a mouse model of depression [92]. In contrast, luminal 5-HT alters the gut bacterial population. Exposure to 5-HT reduces the expression of sporulation factors and membrane transporters in *Turicibacter sanguinis* [93], and increased enteric 5-HT results in gut dysbiosis characterized by increased *Bacilli* species, and decreases in *Bifidobacterium* species and *A. muciniphila* populations [94]. Low availability of 5-HT alters the gut bacterial composition, while 5-HT both stimulated and inhibited the growth of commensal bacteria in vitro in concentration-dependent and species-specific manners [95]. These results indicate that a certain enteric resident microbiota has bidirectional communication with the host serotonergic system to promote habitation in the intestines. Together, gut resident microbiota and microbial metabolites regulate the host serotonergic pathway that includes EC cells, tryptophan hydroxylase (TPH), serotonin reuptake transporter (SERT), 5-HT receptors, and microbial quorum sensing, which is detailed below (Figure 1, Table 1 and Table 2).

(1) EC cells

The density of EC cells in the gut is modulated by the microbiota or microbiome-derived products including SCFAs, with specificity related to bacterial species [85]. Indeed, the numbers of intestinal EC cells were reduced in GF animal models [96,104], while the administration of FMT with SPF feces increased 5-HT-producing EC cells and M2 macrophages in the GI tract [105]. *Bacteriodes thetaiotaomicron* restores 5-HT^+^ EC cells and shapes EC networks in the GI tract of GF mice by producing acetate, propionate, and succinate [96], and those metabolites stimulate EC cell activity and increase the availability of tryptophan [81,85]. Mucosal inflammation induced by *Trichinella spiralis* increases the number of EC cells along with development of postinfectious (PI)-IBS [101]. *Clostridium ramosum* stimulates host 5-HT secretion and programs the differentiation of colonic intestinal stem progenitors toward a secretory 5-HT-producing lineage [87]. In contrast, *Bifidobacterium pseudolongum* reduces the content of 5-HT in colonic mucosa by reducing EC cells [103]. These observations indicate that EC cells are regulated by specific bacteria and induction of aberrant serotonergic signaling by dysbiosis.

(2) TPH

Two isoforms, TPH1 and TPH2, comprise TPH, a rate-limiting enzyme involved in the biosynthesis of 5-HT that converts L-tryptophan into 5-hydroxytryptophan (5-HTP), a direct precursor of 5-HT [106,107]. TPH1 is expressed in the peripheral tissue such as in the lungs, heart, and kidneys, and intestines, while TPH2 is primarily expressed in the serotonergic neurons of the brain and ENS [106,107,108,109]. In the intestines, TPH1 is mostly located in EC cells, and regulated by resident microbiota and their metabolites [106,107]. Genomewide association study (GWAS) results demonstrated that polymorphisms of the TPH gene are associated with the development of IBS in humans [110,111,112,113], though contradictory clinical results regarding intestinal TPH1 expression in IBS patients were also reported [113,114,115]. In a preclinical study, colonic *Tph1* expression and 5-HT biosynthesis were increased by microbiota-derived SCFAs, such as butyrate and acetate [81]. On the other hand, host genetics related to the serotonergic pathway alter the gut microbial composition, as *Tph1^–/–^* mice have a different gut microbiota than that of *Tph1^+/+^* mice, which is related to colitis susceptibility [95]. These findings indicate bilateral communication in microbiota-serotonergic pathways related to TPH regulation.

(3) SERT

A member of the neurotransmitter-sodium symporter family termed SERT regulates the extracellular availability of 5-HT in the gut and brain by 5-HT uptake [116]. Gut mucosal SERT expression is regulated by multiple stimulation factors, including glucagon-like peptides, transforming growth factor beta, immune response, inflammation, growth factors, and microbiota members [116]. A genetic or environmental abnormality in SERT expression is associated with aberrant mucosal 5-HT levels, and can cause a variety of GI functional diseases including IBS [116,117]. *T. spiralis* and *Campylobacter jejuni* reduce SERT expression in the gut [100,101], while *L. rhamnosus* upregulated gene expression and protein levels of SERT in a rat model of PI-IBS [100], and *Lactobacillus acidophilus* and *Bifidobacterium longum* supernatants to upregulate SERT expression in HT-29 and Caco-2 cells [102]. SERT deficiency is related to dysbiosis and changes in the metabolic function of the mouse enteric microbiome [94], and female SERT^–/–^ rats showed visceral hypersensitivity and accelerated GI motility [118]. In humans, reduced SERT expression was observed in the rectal tissue of patients with IBS [119]. GWAS results also indicated that polymorphisms of SERT are susceptible to IBS development [14,120]. On the other hand, an increase in gut luminal 5-HT level by oral supplementation with 5-HT or SERT deficiency in the host increases the relative abundance of spore-forming *T. sanguinis* organisms that uptake 5-HT, which can be reversed by exposure to fluoxetine, a selective serotonin reuptake inhibitor (SSRI) [93].

(4) 5-HT receptors

The 5-HT receptors have a variety of biological functions in the host, such as increasing visceral hypersensitivity and mucosal permeability, inducing inflammation along with activation of immune cells, and changing gut motility [28]. These wide-ranging effects may be because of the vast localization and diversity of 5-HT receptors. So far, 14 different 5-HT receptors in 7 families (5-HT_1–7_) were identified [121], with 5-HT_3_ and 5-HT_4_ being the most investigated serotonin receptors in the intestine. The 5-HT_3_ receptors are present in sensory and myenteric neurons, while 5-HT_4_ receptors are located in presynaptic sites [122]. Agents targeting these receptors are commonly utilized in clinical practice for treating IBS patients [7,123]. The function and expression of 5-HT receptors are also regulated by gut resident microbiota. Normal gut microbes increase colonic 5-HT production and activate the 5-HT_4_ receptor [124,125], while *A. muciniphila* increases the gene expression of *Htr4*, and decreases that of the *Htr2B, Htr3B,* and *Htr7* genes in the colon [89]. The colonization of GF mice with spore-forming bacteria also increased colonic 5-HT by the upregulation of *Htr4* [85].

(5) Quorum sensing (QS)

5-HT is critical for both host physiological functions and communication among gut microbiota members. Additionally, it plays a significant role in QS, as it enables bacteria to detect and control cell population density by releasing small molecules and altering their gene expression [126]. Under homeostatic conditions, 5-HT-dependent QS can be helpful in forming a steady microbial flora, and prevent pathobiont invasion and settlement, while an abnormal QS process based on the dysregulation of 5-HT signaling potentially induces a dysbiosis condition in patients with IBS. QS-regulated mediators produced by *Staphylococcus aureus* interact with intrinsic intestinal neurons and smooth muscle cells, and cause dysmotility in the host GI system [127], a process potentially involved in the pathology of PI-IBS [128]. Furthermore, 5-HT activates virulence factors in and biofilm formation by pathogenic bacteria through QS [129].

## 4. Microbiota-Mediated Serotonergic Signaling in IBS Pathology

**Figure 2:**
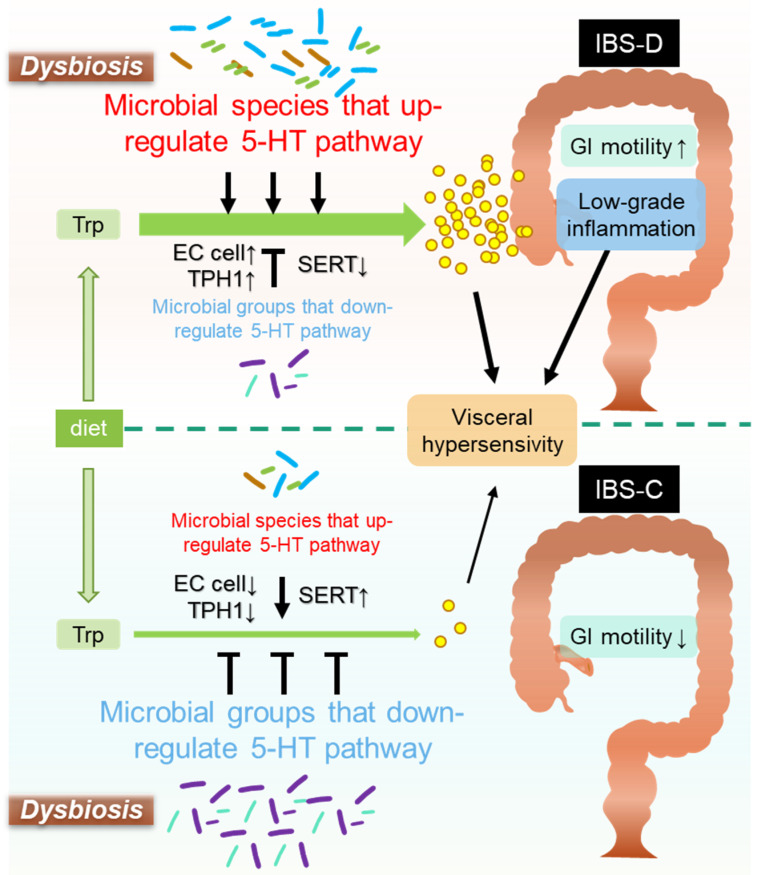
Influence of gut dysbiosis on serotonergic signaling in pathology of IBS. A dysbiotic condition with abundant microbial species that upregulate 5-HT pathway and/or diminished microbial groups that downregulate 5-HT signaling results in massive synthesis and hyperavailability of 5-HT through activating enterochromaffin (EC) cells and tryptophan hydroxylase 1 (TPH1), and decreasing expression of serotonin reuptake transport (SERT). Excessive 5-HT can induce low-grade mucosal inflammation, enhance visceral pain sensation, and promote GI motility, potentially leading to IBS-D development. In contrast, the other dysbiotic condition with decreased microbial species that upregulate 5-HT pathway and/or increased microbial groups that downregulate 5-HT signaling causes limited production of 5-HT, resulting in impaired GI motility. This condition may be associated with IBS-C or chronic constipation. Trp, L-tryptophan; IBS-D, diarrhea-predominant irritable bowel syndrome; IBS-C, constipation-predominant irritable bowel syndrome.

The enteric-microbiota-mediated serotonergic pathway plays a homeostatic role in GI functions [28], while dysfunction of the pathway may contribute to shaping IBS features. We now discuss the molecular mechanisms of the microbe-mediated serotonergic system related to IBS features, including GI motility, visceral pain sensation, and mucosal inflammation with activated immune response (Figure 2).

(1) Role of Gut Microbe-Mediated 5-HT Signaling in GI Motility

The role of 5-HT in GI motility was extensively investigated [130]; 5-HT stimulates peristaltic reflexes in the GI tract, resulting in ascending contractile and descending relaxant limbs [83], while it regulates segmentation motor patterns in the small intestine of guinea pigs [131]. Serotonergic neurons appear to be more important than EC cells are for the regulation of constitutive GI motility, as *Tph2^–/–^* but not *Tph1^–/–^* mice showed delayed GI motility, which is due to reductions in contractile complexes and excitatory synaptic transmission associated with low 5-HT availability [132]. In addition, *Tph2^–/–^* mice have an immature ENS, particularly dopaminergic neurons, responsible for homeostatic GI movement [132]. Mice with the SERT Ala56 mutation have hyperactive SERT function and low 5-HT availability, resulting in decreased intestinal motility shown in both in vivo and in vitro findings, which can be reversed by 5-HT_4_ receptor antagonists [133]. The use of the SERT antagonist fluoxetine enhances GI motility and SERT^–/–^ mice with hyperavailability of 5-HT also show such enhanced motility [133]. Furthermore, endogenous 5-HT can function as a modulator of GI motility via activation of the 5-HT_3_ and 5-HT_4_ receptors in the ENS [134], while antagonists of those receptors was reported to cause a delay in intestinal transit and reverse corticotrophin-releasing hormone-induced defecation in rats [135,136].

Enteric resident microbiota play a key role in serotonergic pathway-mediated GI motility. Indeed, GF animals show significantly slower GI transit as compared to control animals with normal gut microbiota, while treatments with antibiotics delay GI motility along with decreased peripheral 5-HT biosynthesis [137]. Other reports showed that colonization with gut microbiota increases gut motility in ex-GF animals in conjunction with an elevated luminal 5-HT level, while administration of pharmacologic antagonists of 5-HT_4_ receptors resulted in recovery GI transit in GF rodents [105,124,138]. In addition, gut bacteria-derived 5-hydroxyindole, a major 5-HT metabolite, translocates through intestinal smooth muscle cells and directly accelerates colonic motility via the activation of L-type calcium channels [139]. Moreover, BTBR mice, a mouse model of ASD, showed an impaired serotonergic pathway with the downregulation of *Tph1* and upregulation of *Sert* in the gut, which was associated with a reduction in 5-HT-producing *Blautia* bacteria [140]. BTBR mice also demonstrated impaired bile acid synthesis due to a decrease in bile-metabolizing *Bifidobacterium* and *Blautia* bacterial species in the gut, which causes increased mucosal permeability, delayed GI transit, and an autism-like behavioral phenotype [140]. Furthermore, 5-HT with gut microbial stimulation can increase the number of M2 macrophages adjacent to the ENS in the colonic muscular layer, known to be associated with acceleration of GI motility [105]. SCFAs, microbial metabolites, stimulate colonic transit via intraluminal 5-HT release, potentially through the GPR43 receptor on mucosal mast cells [141,142]. Together, these findings show that enteric microbial members regulate GI motility through 5-HT signaling by multiple mechanisms, while dysregulation of this system causes aberrant GI movement, potentially related to an IBS symptom.

(2) Role of Gut Microbe-Mediated 5-HT Signaling in Visceral Pain Sensation

Commensal microbiota activities are critical for developing homeostatic pain sensitivity by inducing normal excitability in the gut sensory neurons, which are absent in GF animals. Indeed, while GF mice show limited mucosal inflammation, visceral hypersensitivity due to altered pain processing in the brain is evident, which can be normalized by FMT with feces from conventional mice [143,144,145]. Gut microbe components, such as certain TLR ligands, formyl peptide receptor 1 agonists, and SCFAs, can directly enhance visceral pain sensitivity by stimulating primary nociceptive neurons in dorsal root ganglia (DRG) or indirectly by activating inflammatory immune response in the gut [146,147]. On the other hand, microbe-mediated kynurenic acid, serine proteases, and bile acids directly reduce pain by inactivating DRG neurons or indirectly by releasing opioid-like factors from mucosal immune cells [146].

The gut microbiota also plays a significant role in development of chronic abdominal pain, an essential feature of IBS [1,2,147,148]. Fecal microbiota samples obtained from IBS patients transmitted hypersensitivity to colonic distension in rats, indicating that gut microbial components are responsible for abnormal pain sensation associated with IBS [149]. In addition, gut-microbiota-mediated neurotransmitters play a significant role in visceral pain sensation [18,22]. Among those neurotransmitters, peripheral 5-HT appears to preferentially act to induce pain by stimulating mesenteric sensory fibers, and vagal and spinal afferent fibers [136]. The release of enteric 5-HT is associated with the severity of abdominal pain in IBS patients, though the effect of 5-HT is dependent on type of 5-HT receptors activated [82,121]. The 5-HT_3_ receptor located in vagal afferent nerve endings in the gut and peripheral endings in spinal afferent nerves plays an important role in visceral hypersensitivity and the nociceptive process as part of the pathogenesis of IBS [29,150]. The central terminals of vagal afferents also exhibit the 5-HT_3_ receptor, which modulates other neurotransmitters in the brain [147,150]. As a result, the activation of that receptor promotes the excitability and activity of GI vagal afferents, while ramosetron, an antagonist of the 5-HT3 receptor, effectively reduces visceral hypersensitivity and modulates GI transit in IBS-D patients [7,150,151]. Moreover, microbiota-mediated mucosal inflammation and increased permeability in the gut is a trigger of pain through the 5-HT pathway, while 5-HT_3_ receptor antagonists have an anti-inflammatory role [152]. Increased mucosal permeability in PI-IBS promotes intestinal mechanosensitivity, which has an effect on visceral sensitivity. Indeed, 5-HT metabolism dysfunction in IBS patients is associated with deterioration of intestinal barrier function [152,153,154]. Together, these results indicate that the gut microbiota is deeply involved in 5-HT-mediated pain sensations, while a dysfunction of the serotonergic pathway associated with enteric dysbiosis in IBS cases can cause visceral hypersensitivity.

(3) Role of Gut-Microbe-Mediated 5-HT Signaling in Mucosal Inflammation and Immune Response

The 5-HT promotes intestinal inflammation through various 5-HT receptors on a variety of mucosal immune cells [155,156]. Indeed, increased mucosal 5-HT and EC cell numbers have been observed in association with several inflammatory conditions including IBD. In animal models of colitis, 5-HT activates peritoneal macrophages and also splenic dendritic cells to produce proinflammatory cytokines in a nuclear factor kappa B-dependent manner, along with sequential T cell activation [157,158]. *Tph1^–/–^* mice with low 5-HT availability are tolerant to experimental colitis [95], while SERT-deficient animals with elevated 5-HT availability were susceptible to gut mucosal inflammation [159]. The pharmacological inhibition of enteric 5-HT by peripheral TPH inhibitors alleviates inflammation [160]. These findings indicate that enteric 5-HT plays a proinflammatory role in intestinal inflammation.

Persistent low-grade mucosal inflammation with aberrant immune cell activation after development of severe enterocolitis is one of the mechanisms underlying the pathogenesis of IBS, especially PI-IBS [161,162,163]. A massive infiltration of mast cells in colonic mucosa associated with the elevated availability of mucosal 5-HT was observed in IBS patients and is correlated with abdominal pain [164]. Although it remains unknown why mucosal inflammation persists even after elimination of pathobionts, the dysbiosis of gut resident microbiota and altered serotonergic signaling are key factors for PI-IBS development. For example, helminth *T. spiralis* induced PI-IBS in mice, along with increased luminal 5-HT level and numbers of 5-HT^+^ EC cells, and reduced SERT expression in the gut [165]. Chemically induced colitis promotes visceral hypersensitivity by increasing the number of 5-HT_3_ receptors expressing nerve fibers in the GI tract [166]. Hypersensitivity to 5-HT in colonic serosal and mesenteric endings remains after recovery from colitis because of persistent alterations in the dynamics of enteric 5-HT_3_ receptors and mast cells [167]. Obtained findings with a postinflammatory IBS rat model showed visceral hypersensitivity accompanied by fecal microbial dysbiosis, elevated serum 5-HT level, the upregulated expression of colonic 5-HT_3A_/5-HT_2B_ receptors, and impaired tight-junction protein expression, while the administration of a 5-HT_3A_ receptor antagonist or FMT from the feces of normal healthy rats alleviated the IBS-like symptoms [168]. In addition, microbiota-derived SCFAs play an important role in maintaining gut homeostasis, and appear to serve a dual role in GI mucosal immunity and inflammation [169]. They play an anti-inflammatory role by strengthening epithelial-barrier integrity through the upregulation of G-protein coupled receptors in the gut, and inducing and maintaining regulatory T cells [170,171]. On the other hand, SCFAs can induce mucosal inflammation through the upregulation of TPH1 transcription and the promotion of mucosal 5-HT production in the serotonergic pathway [81]. Together, these findings show that gut microbial dysbiosis is associated with persistent low-grade mucosal inflammation accompanied by aberrant serotonergic signaling in IBS.

## 5. Microbiota Mediation of Serotonergic Signaling Outside GI Tract

The role of 5-HT was initially investigated in regard to brain homeostasis and psychiatric disorders [172,173,174]. Central 5-HT influences various CNS-related activities, such as nociception, sleep, sexual behavior, cognition, reward, learning, memory, emesis, motor tone, and body temperature in a homeostatic condition [172,173,174]. Furthermore, certain populations of patients with psychiatric diseases, including depression and anxiety, demonstrate aberrant serotonergic signaling in the brain and are treated by drugs targeting 5-HT pathways [175,176]. On the other hand, accumulating evidence indicates that enteric microbiota and its metabolites are essential in maintaining brain homeostasis through the serotonergic pathway, while gut dysbiosis is deeply associated with CNS disorders through the dysregulation of 5-HT signaling in the BGM axis [44,177,178]. Manipulation of the gut microbiota can be effective in treating CNS diseases that regulate 5-HT level as one of the mechanisms [179]. Since IBS and neurological diseases are often complicated, and treatment targeting 5-HT signaling can be effective for both, a dysfunction of the same pathway related to serotonergic signaling could be present in affected patients [180,181].

In the most recent decade, multiple preclinical and clinical studies showed new functions of gut-derived 5-HT in metabolic conditions, such as regarding glucose homeostasis, lipid metabolism, and bone density, and metabolic diseases including obesity and Type 2 diabetes [134]. *T. sanguinis* with a SERT-like structure decreases serum triglyceride level and alters gene expression regarding lipid metabolism, which can be blocked by the use of the SSRI fluoxetine [93]. *Clostridium ramosum* also altered gene expressions related to lipid transport and storage function in mice fed a high-fat diet by increasing *Tph1* expression, 5-HT level, and EC cell number in the gut [87]. Studies that employed pharmacological inhibition or genetic depletion of TPH1 also demonstrated control of glucose homeostasis by enteric microbiota members through regulation of EC cell 5-HT synthesis [182]. A population-based cross-sectional study also indicated that IBS is positively related to metabolic syndrome [183], while integrated and longitudinal multiomics analysis results revealed purine metabolism as a specific host-microbial metabolic pathway in IBS patents [20]. These findings show that an aberrant metabolic pathway is involved in the pathology of IBS, in which certain gut-microbiota components influence host metabolism in a 5-HT-dependant manner. The details of 5-HT-mediated metabolic changes in the pathogenesis of IBS require clarification in future investigations.

## 6. Treatment of IBS by Modulating Microbiota-Mediated Serotonergic Pathways

Pharmacological agents targeting serotonergic pathways, including 5-HT_3_ agonists, 5-HT_4_ antagonists, SSRI, tricyclic antidepressants, and serotonin-norepinephrine reuptake inhibitors, are commonly used to treat IBS patients in clinical practice [7,8,123,181]. Additionally, intervention of the gut microbiota by use of probiotics, prebiotics, synbiotics, elimination diets, and nonsystemic antibiotics, and FMT can be effective in certain IBS cases [6,49,50,51,55,58,184]. However, details regarding microbe-based serotonergic targeting treatment remain largely unknown. An important question is whether specific antibiotics or phage therapy targeting select microbiota can be used to regulate 5-HT and/or its downstream pathway. In terms of FMT, which fecal components can be used to modulate the 5-HT pathway in IBS patients? Is it possible to develop new biomarkers that can efficiently reveal an aberrant 5-HT pathway in IBS patients? Answers to these key questions could shed light on new directions for preclinical research and clinical practice.

## 7. Conclusions

Accumulating evidence indicates that aberrant 5-HT pathways associated with an altered enteric microbiota are deeply involved in the pathogenesis of IBS. However, results remain insufficient to elucidate the whole features of IBS. Particularly important in regard to IBS pathology, it has not been determined whether abnormal serotonergic signaling provides a causal effect related to dysbiosis or is a consequence of an alternating microbial community. Novel findings based on mechanistic studies and large-scale clinical trials regarding microbe-mediated serotonergic pathway are required to fully uncover the pathogenesis of IBS, which could be helpful to provide better treatment for IBS patients.

## Figures and Tables

**Table 1:** Specific enteric microbiota members that regulate serotonergic pathway in GI.

**Upregulation of 5-HT in** **Microbiota**	**Mechanisms of Action and Observations**	**Ref.**
*Akkermansia muciniphila (Amuc_1100)*	Promote intestinal 5-HT biosynthesis and extracellular availability through TLR2 signaling.	[88]
*Akkermansiamuciniphila* (extracellular vesicles)	Increase expression of the *Htr4* gene, and decreases that of the *Htr2B, Htr3B,* and *Htr7* genes.	[89]
*Bacteriodes thetaiotaomicron*	Restore 5-HT^+^ EC cells and shape EC networks in the GI tract of GF mice by producing SCFAs.	[96]
*Bifidobacterium dentium*	Increase intestinal 5-HT level, expressions of 5-HTra receptors 2a and 4, and SERT by producing acetate.	[97]
*Clostridium ramosum*	Promote 5-HT synthesis in colonic EC cells and program differentiation of intestinal stem progenitors toward a secretory 5-HT-producing lineage.	[85,87]
*Corynebacterium* spp., *Enterococcus* spp., *Streptococcus* spp.	Enable the direct production of 5-HT.	[86]
*Escherichia coli Nissle 1917*	Enhance 5-HT bioavailability in ileal tissue through interaction with compounds secreted from host tissue.	[91]
Indigenous spore-forming bacteria	Enhance colonic 5-HT pathway by upregulation of *Htr4.*	[87]
*Lactobacillus plantarum IS-10506*	Increase gut 5-HT production along with brain 5-HTT, neurotrophin, and brain-derived neurotrophic factor.	[98]
*Lactobacillus plantarum PS128*	Increase 5-HT^+^ cells in the gut and alter expression levels of *Tph1*, *Chga*, *Slc6a4*, and *Htr4.*	[99]
SadA-expressing *Staphylococci*	Promote converting 5-HTP into 5-HT.	[90]
*Trichinella spiralis and Campylobacter jejuni (pathogens)*	Increase EC cell number and reduce SERT expression.	[100,101]
**Downregulation of 5-HT in Microbiota**	**Mechanisms of action and observations**	**Ref.**
*Bifidobacterium longum* and *Lactobacillus acidophilus*	Upregulate SERT expression.	[102]
*Bifidobacterium pseudolongum*	Diminish EC cells.	[103]
*Lactobacillus rhamnosus*	Upregulate gene and protein level of SERT.	[92,100]

**Table 2:** Specific enteric microbiota influenced by aberrant serotonergic pathway in GI.

5-HT Pathway-Induced Specific Dysbiosis	Mechanisms of Action and Observations	Ref.
*Turicibacter sanguinis*	Reduce sporulation factors and membrane transporters by 5-HT supplementation and in SERT^−/−^ mice.	[93]
*Bacilli* spp. (*Lactobacillus, Streptococcus, Enterococcus*, and *Listeria*)	Increase in SERT^−/−^ mice.	[94]
*Bifidobacterium* spp.	Decrease in SERT^−/−^ mice.	[94]
*Akkermansia muciniphila*	Increase in *Tph1^–/–^* mice and decrease in SERT^−/−^ mice.	[94,95]
*Two distinct Bacteroidales OTUs*	Decrease in *Tph1^–/–^* mice.	[95]
